# Giant ‘asymptomatic’ ascending aortic aneurysm

**DOI:** 10.1007/s12471-023-01767-2

**Published:** 2023-03-10

**Authors:** Jules R. Olsthoorn, Ka Yan Lam

**Affiliations:** Department of Cardiothoracic Surgery, Catharina Hospital, Eindhoven, The Netherlands

A 76-year-old female consulted her general practitioner because of new-onset leg oedema and dyspnoea. Medical records revealed a history of smoking and hypertension. The diagnosis of chronic obstructive pulmonary disease was considered, and a chest X‑ray was performed, which showed a broadened mediastinum. Computed tomography angiography revealed a supracoronary aorta aneurysm of 10.1 cm in diameter reaching to the brachiocephalic trunk (Fig. 1 and see Video 1 in Electronic Supplementary Material). The patient was accepted for urgent aortic surgery. After induction of anaesthesia and during preparations for cardiopulmonary bypass, multiple episodes of hypotension with desaturation occurred, which were treated with fluid administration. During hypothermic circulatory arrest with selective cerebral perfusion, the ascending aorta and arch were replaced.

**Fig. 1:**
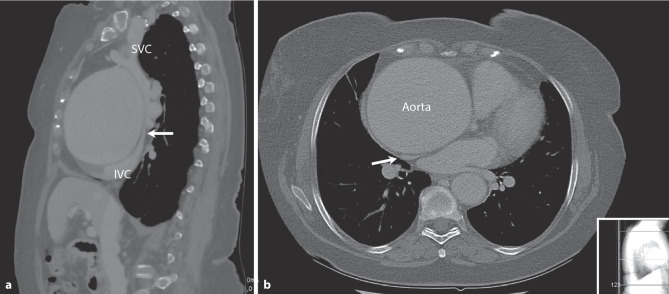
Computed tomography angiography of aorta, in **a** sagittal view and **b** transversal view. *Arrows* mark compression of both superior vena cava (*SVC*) and inferior vena cava (*IVC*)

An isolated supracoronary aorta aneurysm larger than 10 cm is rare [1] and is mostly of unknown aetiology. In retrospect, this large aneurysm caused compression of the left atrium and the inferior and superior vena cava, compromising cardiac preload, mimicking cardiac tamponade and causing pulmonary congestion and chronic venous stasis, which explained the patient’s symptoms.

## Supplementary Information

**Video 1** Three-dimensional reconstruction of thoracic and abdominal aorta
